# Iodide Functionalized Paper-Based SERS Sensors for Improved Detection of Narcotics

**DOI:** 10.3389/fchem.2021.680556

**Published:** 2021-09-08

**Authors:** Li-Lin Tay, Shawn Poirier, Ali Ghaemi, John Hulse, Shiliang Wang

**Affiliations:** ^1^ National Research Council Canada, Metrology Research Centre, Ottawa, ON, Canada; ^2^ Defense Research and Development Canada, Suffield Research Centre, Medicine Hat, AB, Canada

**Keywords:** SERS, opioids, fentanyl, heroin, narcotics, iodide functionalization, sensor

## Abstract

An inkjet-printed paper-based Surface-enhanced Raman scattering (SERS) sensor is a robust and versatile device that provides trace sensing capabilities for the detection and analysis of narcotics and drugs. Such sensors generally work well for analytes with good binding affinity towards the Au or Ag plasmonic nanoparticles (NPs) resident in the sensors. In this report, we show that iodide functionalization of the printed sensors helps to remove adsorbed contaminants from AuNP surfaces enabling superior performance with improved detection of narcotics such as fentanyl, heroin and cocaine by SERS. SERS signals are easily doubled with the iodide-functionalized sensors which also showed orders of magnitude improvement in detection limit. In this report, we show that a short (90 s) iodide treatment of the sensors significantly improved the detection of heroin. We propose that iodide functionalization be integrated into field detection kits through the solvent that wets paper-based sensor prior to swabbing for narcotics. Alternatively, we have also demonstrated that iodide functionalized sensors can be stored in ambient for up to 1 week and retain the improved performance towards heroin detection. This report will help to significantly improve the performance of paper-based sensors for field detection of narcotic drugs.

## Introduction

Timely detection and identification of chemical and biological hazards is a critical challenge faced by first responders of many different professions. Raman spectroscopy is an information rich technique that offers non-invasive chemical identification capabilities and has found many applications ranging from narcotic drug analysis to environmental monitoring. Among the many portable analytical instruments, handheld Raman analyzers have become quite common. (Crocombe, 2018) Most handheld Raman instruments are equipped with an onboard spectral identification function that allows rapid identification of chemical species in the field. Unfortunately, Raman spectroscopy is limited by its poor sensitivity due to the inherently small scattering cross-sections of most molecules. Surface Enhanced Raman Spectroscopy (SERS) bridges this gap and offers unparalleled sensitivity for the detection and identification of an equally broad range of chemical and biological species. (Clarke et al., 2017; Milliken et al., 2018; Langer et al., 2020) SERS has found applications covering security (Wang et al., 2016), food safety (Tay et al., 2012; Drake et al., 2013; Lynk et al., 2018), environmental sensing (Jones et al., 2009; Castro-Grijalba et al., 2020) and most notably bio-chemical analysis. (Huang et al., 2009; Zheng et al., 2018) Most of these tests are designed as laboratory based analytical studies. However, it is a mature technique and coupled with the availability and continual advances in handheld Raman analyzers, SERS is well suited for many field detection challenges.

Since its discovery SERS (Fleischmann et al., 1974; Albrecht et al., 1977; Jeanmaire and Van Duyne., 1977) has now been applied in countless applications. SERS substrates designed and engineered with plasmonic nanostructures have taken on many different forms. Those based on the ubiquitous colloidal Ag or Au sols are still the most popular. The SERS effect is largely due to the collective excitation of the localized surface plasmon resonance sustained by plasmonic nanoparticles (NPs). (Moskovits, 1978; Moskovits et al., 2002) The resulting intense and highly localized electromagnetic field is most pronounced in the inter-particle junction of tightly coupled NPs. Molecules that lie near or in the interparticle junctions (often termed plasmonic nanocavities) benefit from this large field enhancement and manifest as the intense SERS observed in the far-field measurements. (Halas et al., 2011; Tay and Hulse., 2013)

Paper-based SERS sensors are robust and versatile devices that provide trace detection capabilities. (Yu and White, 2013; Haddad et al., 2018) They can be fabricated easily by directly depositing colloidal Ag or Au NPs onto paper-based substrate. Deposition can be done in many different ways, drop-casting, filtering, spray coating or inkjet-printing. Recently, we have demonstrated fabrication of paper-based SERS sensors through inkjet printing of custom colloidal NP ink onto filter paper substrates. (Tay et al., 2021) This type of paper-based SERS sensor provides the point-of-need sampling capability that can be readily used with most handheld Raman analyzers. While SERS sensors generally work well with analytes that either have a directly binding moiety (such as a thiol functional group) or affinity towards the plasmonic surfaces, analysis and detection of non-binding molecules can be much more challenging. The observed SERS signal comes predominantly from aggregated nanoclusters or more precisely from the interparticle hot-spots that the clusters contain. SERS is an optical near-field effect. To successfully detect the enhanced Raman active vibrations, a molecular analyte needs to be able to get to the interparticle junction, the plasmonic hot-site. Unfortunately, as-synthesized colloidal NPs are often coated with a layer of passivating or stabilizing molecules such as citrate. Analytes with weak affinity towards the NP surface are often unable to displace these molecules. In this report we will demonstrate the strategy of iodide ion modification of gold nanoclusters (AuNP) to remove the surface adsorbed species enabling a much higher detection sensitivity for opioids and narcotic drugs. In particular we show that iodide functionalization of the inkjet-printed paper-based SERS sensors can significantly improve the detection of fentanyl, heroin and cocaine.

## Experimental

### Materials

We have purchased nominally 50 and 80 nm AuNP (EM.GC50 and EM.GC80, citrate capped) from commercial source (BBI Solutions) and further process them for inkjet printing. The optical density of the 50 and 80 nm AuNP purchased from BBI Solutions are 1 and 0.89 (at 520 nm), respectively. Stock solutions of fentanyl, heroin and cocaine used in this study were purchased in 1 mg/ml concentration certified reference materials from Cerilliant Corp. Potassium iodide (KI) was purchased from Sigma Aldrich (≥ 99.0%) and used as is. Raman reporter *Trans*-1,2-bis(4-pyridyl)ethylene (BPE) was purchased from Sigma Aldrich (assay 97%).

### Inkjet-Printing of SERS Sensors

To prepare the NP ink formulation for inkjet printing, the as-purchased colloidal Au sol was cleaned and concentrated by centrifugation. The AuNP sol is spun down in a micro-centrifuge, the supernatant decanted and the pellet re-suspended to 1/5^th^ of the original volume. Commercial AuNP purchased from BBI solutions has a manufacturer specified concentration of 4.5 × 10^10^ and 1.1 × 10^10^ for the 50 and 80 nm Au Sol, respectively . The concentration of the 80 nm AuNP ink used for printing is 5.5 × 10^10^ NP/mL while the 50 nm AuNP ink is 2.25 × 10^11^ NP/mL.

For inkjet printing of SERS sensors, we modified a commercial piezoelectric inkjet printer, Epson WorkForce WF-7100, and developed a printing process that is suitable for producing reliable SERS sensors. Empty ink cartridges and ink reservoirs were purchased from commercial suppliers (Ink Owl and Epson distributers, respectively) to accommodate the use of custom nanoparticle ink formulations. SERS sensors were printed in a 0.5 × 0.5 cm square on Whatman 44 filter paper (with 3 µm pore size, 180 µm thickness). For each batch of printing, approximately 12 ml of the concentrated AuNP ink is injected into the empty cartridge. Depending on application, patterns of different dimension and shape are drawn up using the vector graphic editor Inkscape. For printing, filter paper substrates are cut and taped onto a regular piece of letter-sized paper and fed through the printer in a single print run. The printing setup is optimized to maximize the amount of ink deposited in a single print pass. The same sets of sensors are then subjected to multiple printing passes to increase the NP loading in the active sensing area.

### Iodide Functionalization of SERS Sensors

A 0.1 M of KI stock solution was prepared by dissolving KI in DI water. Iodide functionalization was carried out by immersing the printed sensors in a 1 mM KI solution overnight. The sensors were removed from the KI solution, gently rinsed with DI water and allowed to dry in ambient before being exposed them to the appropriate analyte solution.

Fentanyl, heroin and cocaine solutions of concentration ranging from 0.1 mg/ml to 1 ng/ml were prepared by sequential dilution of the 1 mg/ml certified reference material with DI water. SERS sensors with and without iodide functionalization were then immersed in the respective analyte solution for up to 3 h. The sensors were then removed from the analyte solution and allowed to dry in the ambient before Raman measurement.

**FIGURE 1:**
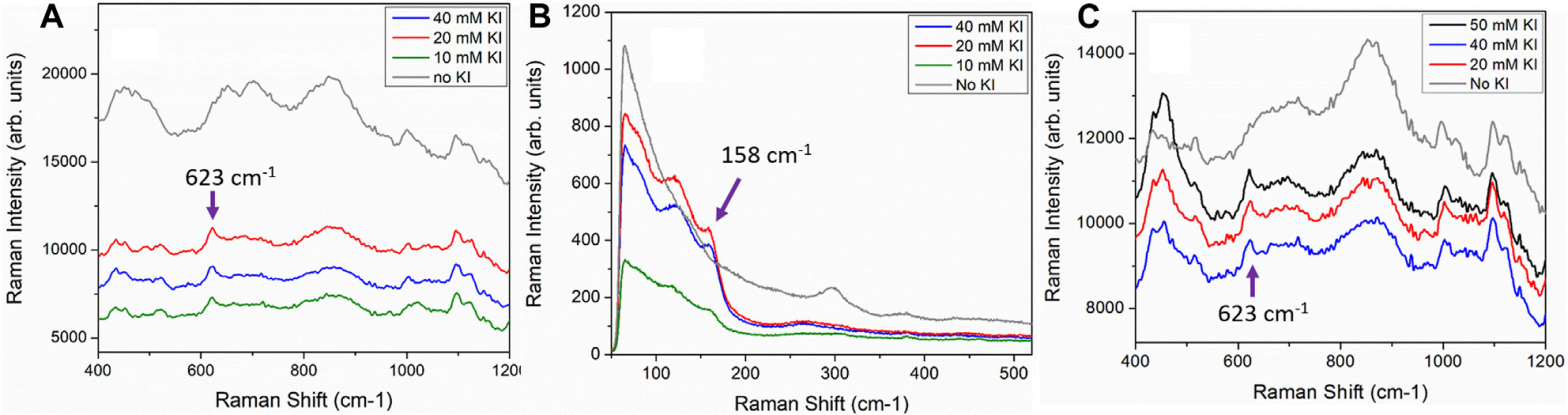
Printed sensors functionalized with 50 mM, 40 mM, 20 mM and 10 mM KI solution for 30 min followed by exposure to a 100 μg/ml heroin solution for 30 min **(A)** SERS spectra acquired with handheld Raman analyzer showing clear heroin peak at 623 cm^−1^. **(B)** Lower wavenumber bands of the sensors shown in A with the iodide functionalized sensors exhibit Au-I band at 158 cm^−1^. SERS spectra acquired with Horiba LabRAM HR microRaman spectrometer. **(C)**. Printed sensors were functionalized with 50 mM, 40 and 20 mM KI solutions for 90 s followed by 90 s of exposure to a 100 μg/ml heroin solution. SERS spectra acquired with handheld Raman analyzer. Control sensors with no exposure to KI prior to heroin immersion are shown as grey spectra in each panel.

In order to study the applicability of iodide treatment for field sensor applications, several sensors were exposed to much higher KI concentrations (50, 40, 20 and 10 mM KI). These sensors were first treated with KI solution for 1.5 min (Figure 1C) to 30 min (Figures 1A,B) followed by immersion in a 100 µg/mL heroin solution for 1.5–30 min. Data used to generate Figure 1 followed this experimental protocol.

It is worth noting that printed SERS sensors remain very stable when immersed in dilute KI solution. We observed no change in colour or performance for printed sensors immersed in 1 or 2 mM solution for over 1 week. However, we did observe that immersion of printed SERS sensors in higher KI concentration (10 mM and above) overnight caused a noticeable color change (fading) most likely due to the detachment of AuNP from the filter paper substrate.

### Instrumentation

SERS sensors were characterized with both a handheld Raman analyzer, ReporteR (SciApps) and a microRaman spectrometer (Horiba Jobin Yvon, LabRAM). The ReporteR is equipped with a 70 mW, 785 nm excitation laser; a 2048 pixel TE cooled CCD array detector and an 1800 lines/mm grating. It covers a spectral range of 300–2,500 cm^−1^ with 12 cm^−1^ spectral resolution. With the right-angle attachment, the laser illumination spot size is approximately 25 µm. The system is connected to a laptop for spectral acquisition and manipulation for all the measurements performed in this study. We use the software Nuspec supplied by the manufacturer to control the spectrometer. Before each experiment, the system is validated and calibrated using the manufacturer supplied polystyrene reference standard and the manufacturer recommended procedure. The microRaman spectrometer was also used to characterize SERS sensors, particularly to probe the low wavenumber regions beyond the range of the ReporteR. The system has a single-stage spectrograph equipped with a TE-cooled CCD detector. Excitation of the sample was done in the retro-reflective (backscattering) geometry through a ×20 objective (Olympus, Na: 0.40) with a 632.8 nm laser.

The scanning electron microscopy (SEM) images of the printed SERS sensors were acquired with the Hitachi SU5000 analytical scanning electron microscope equipped for variable pressure field emission. Imaging was carried out at low vacuum mode with an acceleration voltage of 10.0 kV.

## Results and Discussion

### Paper-Based SERS Sensor

**FIGURE 2:**
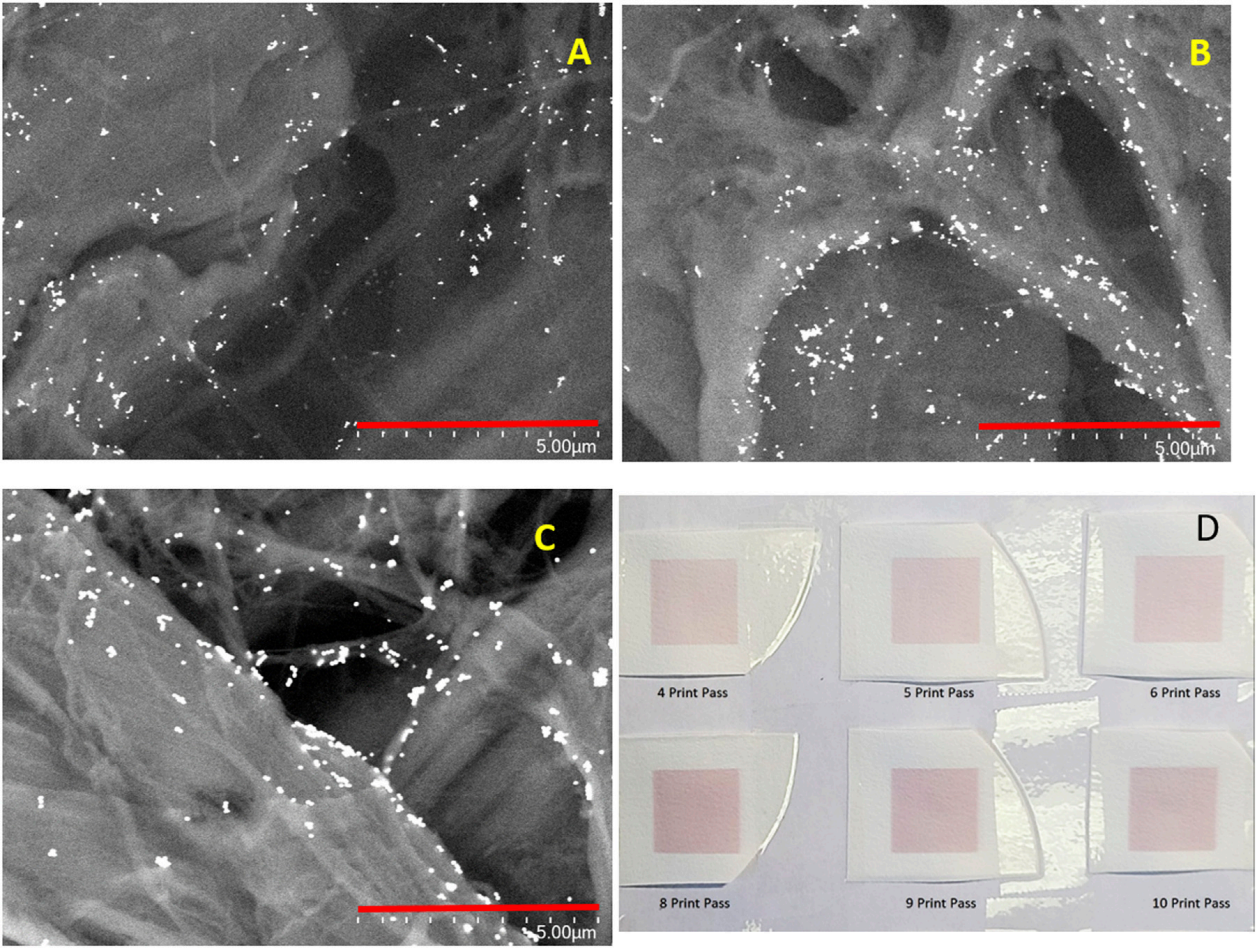
SEM images of SERS sensors printed with **(A)**. 50 nm AuNP and 6-printing passes; **(B)** 50 nm AuNP and 8-printing passes and **(C)** 80 nm AuNP and 8-printing passes. The scale bar is 5 µm in length. **(D)** A photograph of the printed SERS sensors with 4, 5, 6, 8, 9 and 10 printing passes.

Typically, the amount of NP deposited onto the filter paper in one printing pass is insufficient to support strong SERS activity. The number of printing passes required to sustain a strong SERS response was determined empirically and the methodology is detailed in our previous study. (Tay et al., 2021) As the number of printing passes increases, the number of AuNPs deposited onto the filter paper increases as does the aggregate size and the number of aggregates. This is evident from the SEM images shown in Figures 2A,B show the SEM images of sensors printed with 6-printing passes (Figure 2A) and 8-printing passes (Figure 2B) with 50 nm AuNP ink. It is evident that the NP aggregates in Figure 2B appear to be much larger in size and more numerous as compared to the sensors prepared with fewer printing passes (Figure 2A). The image of the 80 nm sensor with 8-printing-passes (Figure 2C) shows large aggregates but with slightly fewer of them as compared to Figure 2B. This is due to the lower concentration of the 80 nm colloidal Au sol, which is approximately 4X less than for the 50 nm Au Sol. A photograph of the sensors printed with 80 nm AuNPs and various printing passes is shown in Figure 2D. As the number of printing passes increases, the colour of the sensors also becomes progressively more saturated.

Inkjet-printed sensors have a number of advantages. The controlled droplet jetting process of a piezo-electric print head produces sensors with a more uniform visual appearance and hence with a more even NP loading across the active area. The inkjet printing process also provides a much more controllable and reproducible procedure for production of a large number of SERS substrates. The controlled jetting process also allows the AuNP in each of the small droplets to wet only a small portion of the cellulose substrates leading to a more even distribution of AuNP on the paper substrates. Although the cellulose substrate has a 3 µm pore, there is no visible loss of NPs through the pores during the printing process. The dark red colour that is clearly visible on the printed side, while the backside of the sensor appears white is evidence that the preponderance of NPs resides on the printed side of the sensor. The majority of the AuNP are retained in the network of cellulose fiber near the surface and very little penetrates through the pores to reach the backside of the paper substrate.

SERS activity of the inkjet-printed paper-based SERS sensor depends on a number of factors such as ink material (e.g., Ag or Au), size of the NPs, and coupling and aggregation state of NPs as well as the affinity of the molecular analyte to the NPs. In the printed sensors, the observed SERS intensity comes predominantly from aggregated nanoclusters. SERS enhancement scales with the size of the AuNPs. Electromagnetic modeling shows that the field enhancement of equilateral gold-trimer aggregates made of three 80 nm AuNPs is almost double that of a trimer made of three 50 AuNPs as shown in supplementary material (Supplementary Figure S1). This near doubling of the field enhancement becomes a greater than 12 times augmentation of the SERS enhancement. Of course, the NP aggregates formed on the cellulose fiber of the paper substrates are a collection of many sizes and shapes of aggregates but the general trend of higher SERS from aggregates made of larger NPs holds. (Moskovits, 2005) We have performed detailed electromagnetic field calculations and measurements of aggregates ranging from 2 to 10 AuNPs. (Tay et al., 2010; Tay et al., 2012) SERS enhancement dependence on the size of the aggregates is in detail more complex but in general, larger aggregates possess many more interparticle junctions which potentially can serve as SERS hot-sites. Our earlier study shows that the field enhancement varies drastically across differing junction hot sites and that enhancement depends strongly on the overall geometry of the nanoaggregates. (Tay and Hulse., 2013) In general, the slightly larger aggregates still provide better enhancement as compared to a simple dimer cluster and significantly greater enhancement than do uncoupled single NPs (monomers).

**FIGURE 3:**
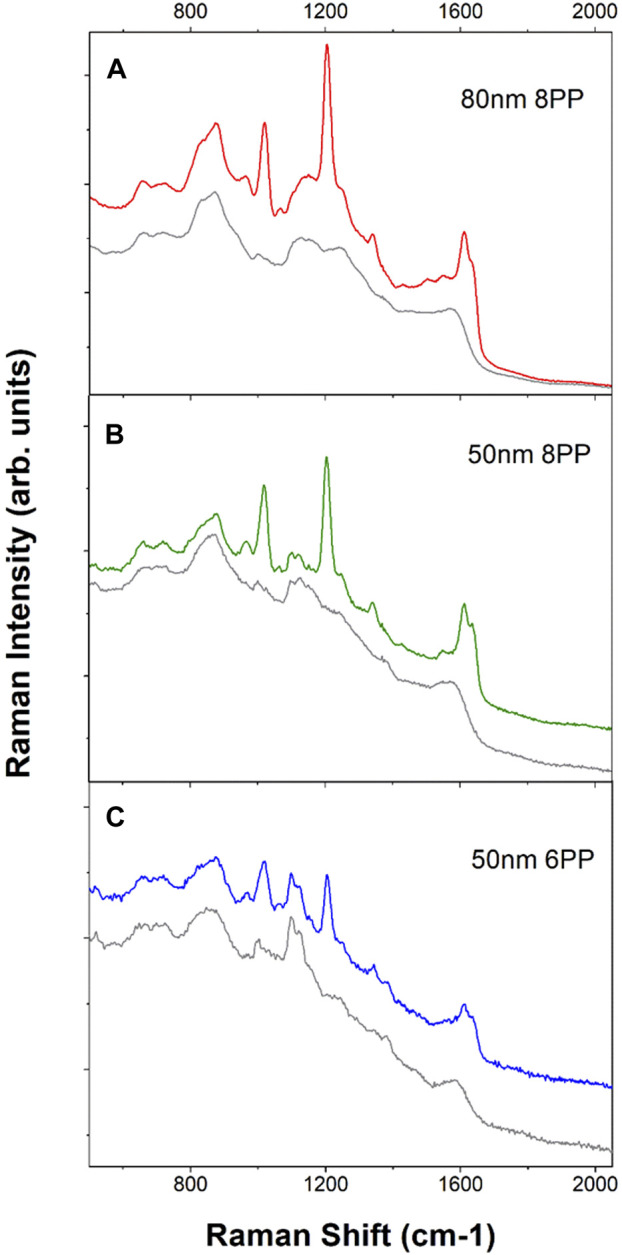
Background (grey traces) and BPE SERS spectra (red, green and blue traces) of the printed sensors. All background spectra were displayed as grey traces. **(A)**. SERS spectra obtained from 80 nm AuNP ink 8-printing passes sensors. The tick increment is 5,000 counts. **(B)**. Spectra from 50 nm AuNP ink 8-printing-passes sensors. The tick increment is 2,000 counts. **(C)**. Spectra from 50 nm AuNP ink 6-printing passes sensors. The tick increment is 1,000 counts. All spectra were acquired with 2 s integration and five accumulations. Each spectrum shown here is the average of spectra obtained from 10 random spots from the printed sensor. All spectra were acquired with handheld Raman analyzer. Spectra have been offset for clarity.

To obtain SERS sensors with sufficient sensitivity for field applications, different sizes of AuNP and printing conditions, specifically, the number of printing passes were tested. The Au nanoaggregates deposited on the filter paper increase both in size and number as the number of printing passes increases. This is evident from the images shown in Figure 2. It also results in higher SERS activities as shown in Figures 3B,C. However, it is also worth noting that our (Tay et al., 2021) and other studies (Hoppmann et al., 2014) have shown that additional printing passes beyond the optimal number of printing passes do not result in a further increase in SERS intensity. In fact, SERS intensity tapers off and even begins to decrease as the number of printing passes increases beyond the optimal value. A Higher number of printing passes also results in a higher SERS background which is not a desirable feature for trace chemical detection. Figure 3 shows the SERS background (grey traces) and SERS (red, green and blue) spectra obtained from sensors produced by eight printing passes of 80 nm (Figure 3A) and 50 nm (Figure 3B) AuNP ink as well as six printing passes of 50 nm AuNP ink (Figure 3C) and then exposed to 1 µM of *trans*-1,2-bis(4-pyridyl)ethylene (BPE). Comparing the two sensors prepared with 50 nm AuNP ink, the sensor with six printing passes (Figure 3C) has a lower background as well as a lower BPE SERS signal than the eight print pass sensor in Figure 3B. The integrated intensity of the BPE 1204 cm^−1^ band is approximately 4.5 times stronger in the eight print-pass sensors as compared to the six print-pass one. It is not surprising that the sensors with lower SERS activities also show a lower SERS background for the “blank” sensors. The AuNP inks are prepared with the standard citrate reduction protocol. The citrate ions that naturally associated to the surface of the AuNPs act as a capping layer and adsorb to the surface of the AuNPs. The SERS spectrum of citrate associated to the citrate reduced AuNP is well characterized and can be seen in the background spectra of “blank” sensors. For example, the weak 1,000 cm^−1^ band associated with bi-dentate citrate ion adsorption on the AuNP surface is visible in all three background SERS spectra from the blank sensors. (Grasseschi et al., 2015; Grys et al., 2020) Here we use the term “blank” to describe the as printed sensors without any surface functionalization or modification. The sensors, as printed, are stored in the ambient. It is possible that other carbonaceous species can then adsorb on the surface of the NPs further contributing to the bands observed in the background spectra. It is also important to point out that a few of the bands observed in the background stem from the contribution of the cellulose substrate, e.g., the features at 1,110 cm^−1^ and 381 cm^−1^. (Wiley and Atalla., 1987; Gelder et al., 2007; Tay et al., 2021)

Aggregates from larger AuNPs also contribute to higher SERS activities as discussed earlier and as demonstrated in the electromagnetic simulation in the Supplementary Figure S1. Comparing the same eight printing passes of SERS sensors made of 80 nm AuNP (Figure 3A) versus 50 nm AuNP (Figure 3B), the integrated BPE SERS intensity at 1,204 cm^−1^ band is approximately 3.5 times larger for the sensor prepared with larger AuNPs. There is an obvious advantage in using larger AuNPs in order to achieve higher sensitivity. However, one would also need to consider the significantly larger background that will be present in the sensors with higher sensitivity. Again, this is not a problem for the detection of analyte molecules such as BPE, which is easily able to displace other weakly adsorbing species near the plasmonic hot-sites. On the other hand, detection of analytes that do not have good affinity towards the AuNP surfaces and trying to determine weak SERS bands on top of a very large background can be extremely challenging. Surface functionalization (or surface modification) is a good way to circumvent the above challenges.

To ensure reproducibility, batch-to-batch and point-to-point SERS uniformity was studied with different batches of sensors treated with BPE analyte. Three different batches of inkjet-printed sensors (batches A, B and C) were prepared with 50 nm AuNPs following the procedure outlined in the experimental *Inkjet-printing of SERS Sensors* . Two sensors from each batch were randomly selected for the batch variation study. Ten spectra from ten random spots were acquired for each sensor. Supplementary Figure S3 shows the relative standard deviation (RSD) of 12% from the six different sensors of the three different printing batches. The RSD indicates good batch-to-batch reproducibility through the well-controlled inkjet-printing process.

### Iodide Functionalization of Printed SERS Sensors

The observed SERS signals from the printed SERS sensors are predominantly from the pre-formed hot-spots from aggregated nanoclusters. Functionalization is a common approach to achieve better sensitivity especially for molecules that do not have a moiety that binds naturally to the Au/Ag surfaces as does a thiol functional group. Because of the limited and restricted volume of the pre-formed hot-spot, it is advantageous to approach functionalization with the smallest possible molecule that will do the job and the iodide ion is a good choice. Compared to other alkyl thiols that have been used as partition layers to improve SERS detection of non-binding molecules, the iodide ion has an ionic radius of 2 Å which is much smaller than the alkyl thios. (Fischer et al., 2003) Its small dimension is particularly advantageous for accessing the extremely confined volume near the pre-formed hot-spots. The small iodide ion can access and potentially displace some of the adsorbed (e.g., carbonaceous) species near the hot-site thus improving the signal-to-noise of the measured analyte signal by reducing interfering background signals.

**FIGURE 4:**
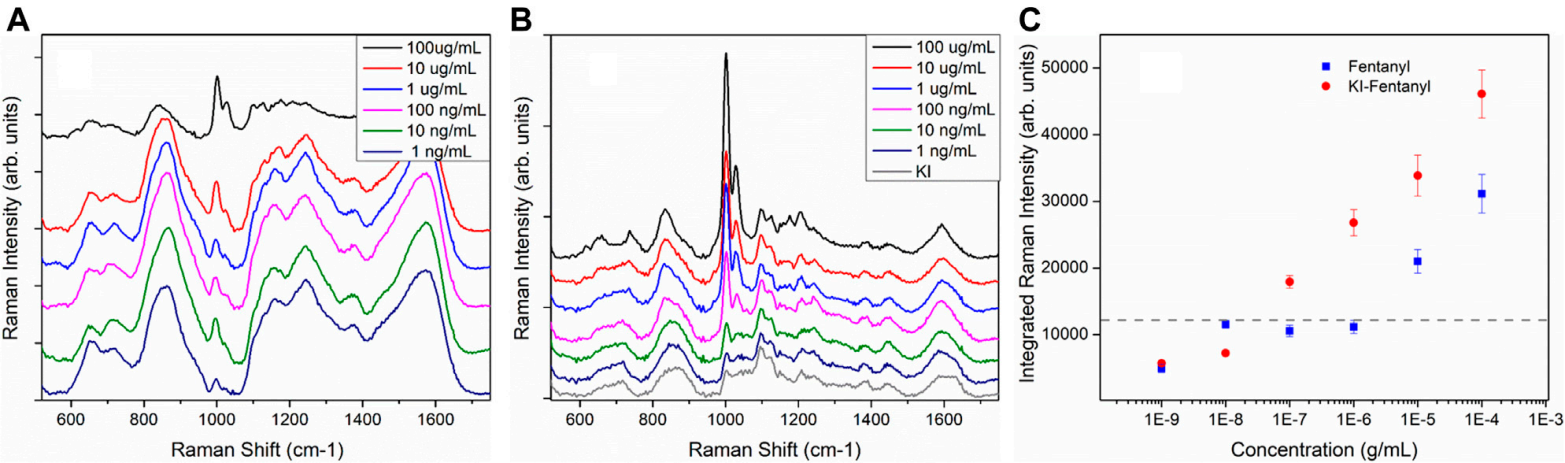
**(A)** SERS spectra of inkjet-printed SERS sensors directly exposed to various concentrations of fentanyl. Spectra have been offset for clarity. **(B)**. The printed sensors are first modified with 1 mM KI followed by exposure to the fentanyl. The grey spectrum shows the SERS background obtained from a sensor exposed to 1 mM KI overnight. Spectra have been offset for clarity. Each of the spectra shown here is an average of 10 spectra acquired from ten random spots on the sensors. All spectra shown here were acquired with ReporteR handheld Raman analyzer. **(C)**. Integrated intensity of the 1,002 and 1,029 cm^−1^ doublet of both sets of sensors. The dashed line shows averaged background intensity obtained from blank sensors. Each spectrum shown here is the average of 10 spectra taken from 10 random spots of each sensor.

Iodide modification for SERS detection was first proposed as a means to obtain a “clean” SERS substrate free from other “impurity” adsorbed species to facilitate label free detection of native protein, nucleic acid and biological molecules. (Huang et al., 2011; Xu et al., 2014; Xu et al., 2015, Zhu et al., 2016). Iodide ion forms a monolayer of AuI bound on the Au surface and readily displaces other adsorbed carbonaceous species, preventing their re-adsorption. The method has been applied to label-free SERS detection of biomolecules. (Huang et al., 2011; Xu et al., 2014; Xu et al., 2015) Zhu et al. has applied the iodide modified pinhole shell-isolated nanoparticle-enhanced Raman spectroscopy (SHINERS) technique for the detection of herbicide. In that paper, the authors also took advantage of iodide functionalization to help replace other impurities by forming an Au-I bond. The iodide ion then further facilitates the electrostatic adsorption of the herbicide to the iodide-functionalized SHINERS substrate thus enabling detection of the herbicide. However, iodide functionalization has not been applied for other label free SERS applications. Field detection of drugs and narcotics is a large application space for SERS sensors that utilize the SERS label-free detection approach. Here we have demonstrated that iodide-functionalization of an inkjet-printed SERS sensor provides significant improvement in the SERS detection of opioids and narcotics. Figure 4A shows the SERS spectra of sensors exposed to various concentrations of fentanyl ranging from 100 µg/ml to 1 ng/ml. Fentanyl vibrations are clearly observable as the doublet at ∼ 1,002 and 1,029 cm^−1^. (Leonard et al., 2017; Haddad et al., 2018) Previous fentanyl SERS studies have suggested that the fentanyl molecule interacts with the metal surface through a C=O (Leonard et al., 2017) terminus and is likely to lie flat on the surface of the NPs. Figure 4B shows the SERS spectra from sensors that were first functionalized with 1 mM of KI followed by exposure of fentanyl solution. These spectra demonstrate significant enhancement of the fentanyl SERS band after iodide functionalization. Figure 4C compares the integrated intensity of the fentanyl band at ∼ 1,002 cm^−1^ of both sensors with and without iodide functionalization. It clearly shows that iodide functionalized sensors (Figure 4C, red-circles) consistently showing higher fentanyl SERS intensities compared to sensors without KI functionalization (Figure 4C, blue-squares). The dashed line in Figure 4C shows the averaged integrated intensity of the blank sensors and provides an indication of the background signal level of the blank sensors over the same spectral range. The detection limit is estimated from the detectable signal above the background level from the lowest analyte concentration. The detection limit of fentanyl for sensor without iodide functionalization is 10 μg/ml. With iodide functionalization, this detection limit improved by two orders of magnitude to 100 ng/ml. This is higher than expected because of the interfering band (at ∼ 1,000 cm^−1^) present in the background of the blank sensor. The detection limit could be improved if this interfering band due to the residual citrate were to be reduced or removed all together.

**FIGURE 5:**
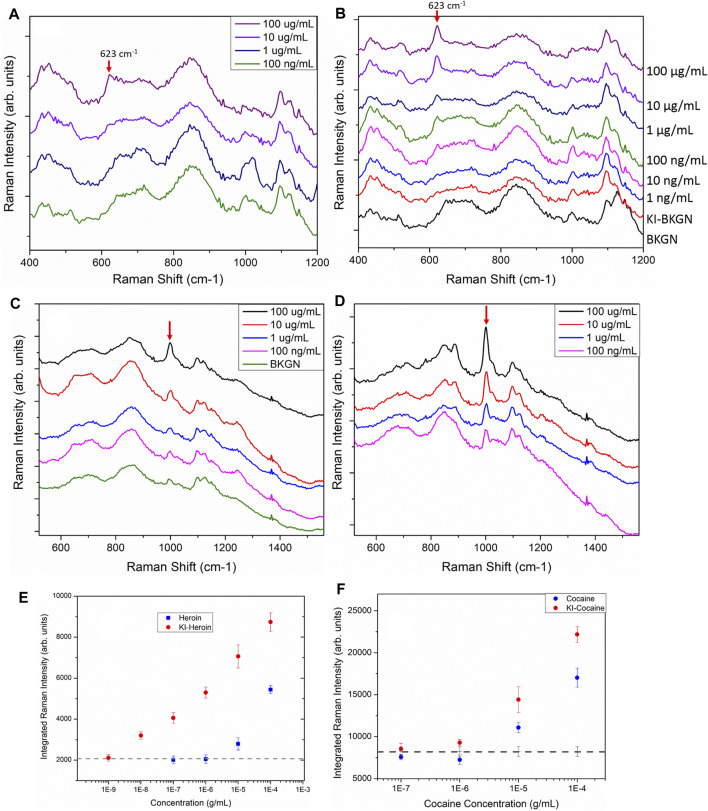
SERS spectra of sensors directly exposed to heroin **(A)** and sensors first functionalized with 1 mM KI before exposure to heroin **(B)**. The red arrows indicate the heroin vibration at 623 cm-1. Spectra have been offset for clarity and the tick increment on the *y*-axis is 2,000 counts. **(C)**. SERS spectra of sensors directly exposed to cocaine of various concentration. The green trace (BKGN) is the spectrum obtained from a blank SERS sensor. **(D)**. Sensors first functionalized with 1 mM KI followed by exposure to cocaine at various concentrations. Arrows indicates the cocaine vibration at 1,002 cm-1. Spectra have been offset for clarity and the tick increment on the *y*-axis is 2,000 counts. All spectra shown here were acquired with the ReporteR handheld Raman analyzer. Each of the spectra shown here is the average of 10 spectra taken from 10 random spots on each sensor. **(E)** The SERS integrated intensity of the 623 cm^−1^ heroin vibrational band for sensors exposed to heroin. **(F)** The SERS integrated intensity of the 1,002 cm^−1^ cocaine vibration from sensors exposed to cocaine. The dashed line in both C and D shows the averaged integrated intensity of the blank sensors and indicates the background signal level.

An even more pronounced improvement is observed using the iodide-functionalized sensors for the detection of heroin. Figures 5A,B shows the heroin spectra obtained from sensors without and with the KI functionalization, respectively. The red arrows indicate the characteristic heroin vibration at 623 cm^−1^. For the sensors without iodide functionalization, the heroin band is observable in sensors treated with a 100 and 10 μg/ml heroin solution (Figure 5A). With functionalization, the characteristic heroin band can be observed down to 10 ng/ml (magenta trace, Figure 5B). A similar trend is observed when spectra of non-functionalized and iodide functionalized sensors are compared after exposure to cocaine as shown in Figures 5C,D. The cocaine band at 1,002 cm^−1^ is much more pronounced for iodide functionalized sensors. The red arrows in Figures 5C,D indicates the position of the ring vibration mode (1,002 cm^−1^) of the cocaine molecule. Without functionalization, this band is clearly above the background signal when the sensor has been exposed to a cocaine concentration of 10 μg/ml or more. With iodide functionalization, the detection limit for cocaine drops to 100 ng/ml.

The SERS integrated intensity of the 623 cm^−1^ band from heroin spectra and 1,002 cm^−1^ from cocaine spectra are shown in Figures 5E,F, respectively. In both cases, sensors functionalized with 1 mM KI before exposure to heroin and cocaine consistently out-performed the sensors without the KI treatment. (Figures 5E,F, red circles). The dashed line in Figures 5E,F shows the averaged integrated intensity of the blank sensors over the same spectral range and provides an indication of the background signal level. Without iodide functionalization, the detection limit of the inkjet-printed SERS sensors are 10 μg/ml for heroin and cocaine. With iodide functionalization, orders of magnitude improvements are observed in the detection limits of heroin (10 ng/ml) and cocaine (100 ng/ml). Similar detection limits (10 ng/ml) of heroin were observed for sensors treated with 10 and 20 mM KI solutions (Supplementary Figure S4). The presence of a distinct background band centered ∼1,000 cm^−1^ (from the residual citrate ions on the AuNPs) interferes with the identification of both fentanyl and cocaine at lower concentrations. The result of this is that for both cocaine and fentanyl, the detection limit is significantly higher than for heroin, where the primary band used for the identification (623 cm^−1^) has no interfering background.

**FIGURE 6:**
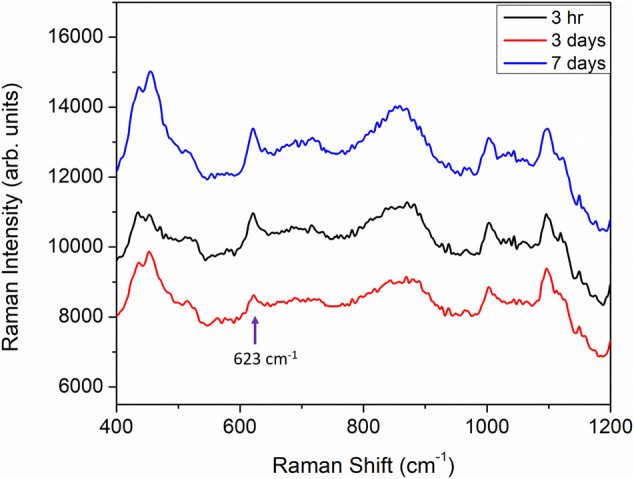
SERS spectra of heroin obtained from sensors functionalized with 20 mM KI for 30 min and exposed to a 100 μg/ml heroin solution for 30 min. After the KI functionalization, the sensors were stored in ambient for 3 h (black trace), 3 days (red trace) and 7 days (blue trace) before heroin exposure.

In the results discussed above, all of the iodide functionalization was carried out by overnight immersion of printed sensors in 5 ml of 1 mM of KI solution. The immersion time in KI solution was typically around 18 h followed by approximately 3 h of exposure of the iodide-functionalized sensors to the analyte solutions. Such long immersion times may not be suitable for field application. We have further tested the sensors with higher concentrations of KI and much shorter incubation times (down to 90 s) to demonstrate field applicability. The iodide functionalization treatment for paper-based sensor is a very simple treatment and it can be implemented easily as part of the field sample collection routine. For example, SERS detection kits are often provided with solvent for wetting of paper-based sensors prior to performing a swipe to obtain a sample. The KI solution can be used for part or all of the sensor wetting procedure prior to swiping of a suspicious surface with the solvent saturated and functionalized sensor. The only apparent drawback of our sensors is that the protocol presented so far requires a rather long functionalization time. This time can in fact be shortened significantly by increasing the concentration of the KI solution to just below 50 mM. Figures 1A,B show three printed sensors functionalized with 40 mM, 20 and 10 mM of KI for 30 min followed by immersion in a 100 μg/ml heroin solution for 30 min. As a control, a printed sensor was immersed directly in a 100 μg/ml heroin solution without KI functionalization for the same duration of 30 min. The sensor without KI functionalization (grey trace in Figure 1) shows almost no-observable heroin signature, whereas the other three sensors functionalized with KI all showed a clear heroin molecular vibration at 623 cm^−1^. The increase in KI concentration will help to drive the reaction in favor of the formation of an Au-I bond thus shortening the necessary immersion time. This immersion time can potentially be shortened even further, with a higher KI concentration. Figure 1C shows the results for sensors treated with 50, 40 and 20 mM KI solutions for 90 s followed by 90 s immersion in a 100 µg/ml heroin solution. All three sensors treated with KI consistently and successfully registered the 623 cm^−1^ heroin band (black, red and blue spectra in Figure 1C), while the control sensor with no KI functionalization did not register any heroin signature (grey spectrum, Figure 1C). This is a compelling result and is fully realizable in a field application setting. It is also important to point out that sensors do not need to be functionalized with KI immediately before use. Figure 6 shows that sensors pre-functionalized with 20 mM KI and stored in ambient for up to 7 days followed by exposure to a 100 μg/ml heroin solution all showed a strong heroin SERS band at 623 cm^−1^. This result shows that sensors can be pre-treated with KI and stored for later use.

Iodide ion forms a strong Au-I bond that is characterized by the Au-I vibration at 158 cm^−1^ (Tang et al., 1998) as shown in the three sensors functionalized with KI in Figure 1B. The control sensor did not exhibit this characteristic Au-I vibrational band at 158 cm^−1^. It is also interesting to point out that the control sensor or sensors without the KI functionalization tends to have much higher backgrounds which is indicative of the carbonaceous contaminants adsorbed on the Au surface. We notice that AuNPs purchased from commercial sources exhibit a prominent low frequency vibration at ∼ 300 cm^−1^. This band disappears after iodide functionalization. This is most likely due to the iodide ion successfully having displaced the other adsorbed specie from the Au surface.

For the results shown in Figure 1, SERS sensors were treated with KI concentration ranging from 10–50 mM. We did not observe any significant improvement in the detection of heroin from the sensors treated with different KI concentrations (Supplementary Figure S4). It is also possible to functionalize the sensor with other halogen ions.Zhu et al. (2016) has assessed the use of other halogen ions but concluded best results were obtained with iodide ion.

## Conclusion

In this paper, we have discussed the physical characteristics of inkjet-printed SERS sensors. Sensors prepared with larger sizes of AuNPs and a higher number of printing passes show better SERS performance. This is not unexpected since the SERS effect typically scales with the size of both the NPs and their aggregates. We have investigated iodide functionalization of the printed SERS sensors. Sensors functionalized with iodide ions generally perform much better by lowering the background contaminants found on untreated NP surfaces. We have shown that sensors treated with KI show a much higher integrated SERS intensity for fentanyl, heroin and cocaine and this translates into an orders of magnitude improvement in the detection limit for all three narcotics. In addition, we have shown that it is possible to shorten the iodide functionalization time to 90 s by increasing the KI concentration. Iodide functionalized sensors are able to detect heroin in a much shorter exposure time of 90 s whereas a control sensor without iodide functionalization did not show an observable heroin signal. We propose that the KI functionalization can be prepared as part of the field detection kit which wets the sensor before swabbing and allows for detection of the narcotic drug with a much higher sensitivity. Finally, we have also shown that sensors that are pre-functionalized and stored over a period of up-to 1 week will still retain their improvement in sensitivity.

## Data Availability

The original contributions presented in the study are included in the article/Supplementary Material, further inquiries can be directed to the corresponding author.
