# Supplementary material for: Iodide Functionalized Paper-Based SERS Sensors for Improved Detection of Narcotics
Source: Front Chem. 2021 Sep 8;9:680556. doi: 10.3389/fchem.2021.680556 (PMC8455876; doi:10.3389/fchem.2021.680556)

Supplementary Material

# Electromagnetic simulation of gold nanoparticle trimers

**Figure S1.** Electromagnetic simulation of two sets of AuNP trimers. (A) is the trimer made of 50 nm AuNP and (B) is the trimer made of 80 nm AuNPs. Excitation is polarized in the x-direction, as indicated by the red arrows. The maximum electric field strengths are 77 and 145 for the 50 nm and 80 nm trimers respectively, located at the interparticle junction of the two NPs at the bottom of the triangle. The trimers are illuminated by a linearly-polarized plane wave incident normal to the plane. The method of calculation is the finite difference time domain, FDTD, numerical technique using codes available commercially from Lumerical Inc. (FDTD: 3D Electromagnetic Simulator). The calculation uses SI units, so electric field strength is in V/m. The Au spheres are configured to be lying in the XY-plane, 2 nm from each other at closest approach.


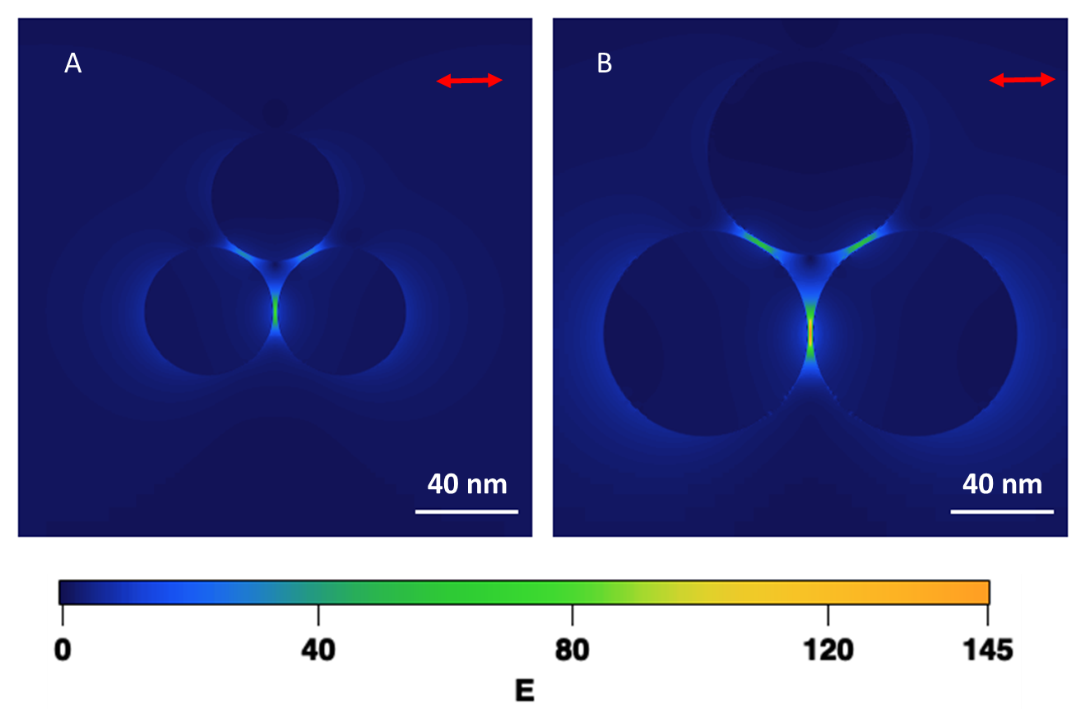


# SEM images of SERS Sensor before and after KI treatment

Figure S2. (a). Scanning electron microscopy images of an inkjet-printed SERS sensor. (b) Image of the same sensor after overnight immersion in a 1 mM KI solution. No obvious loss of AuNPs after the overnight immersion in the KI solution is observed.


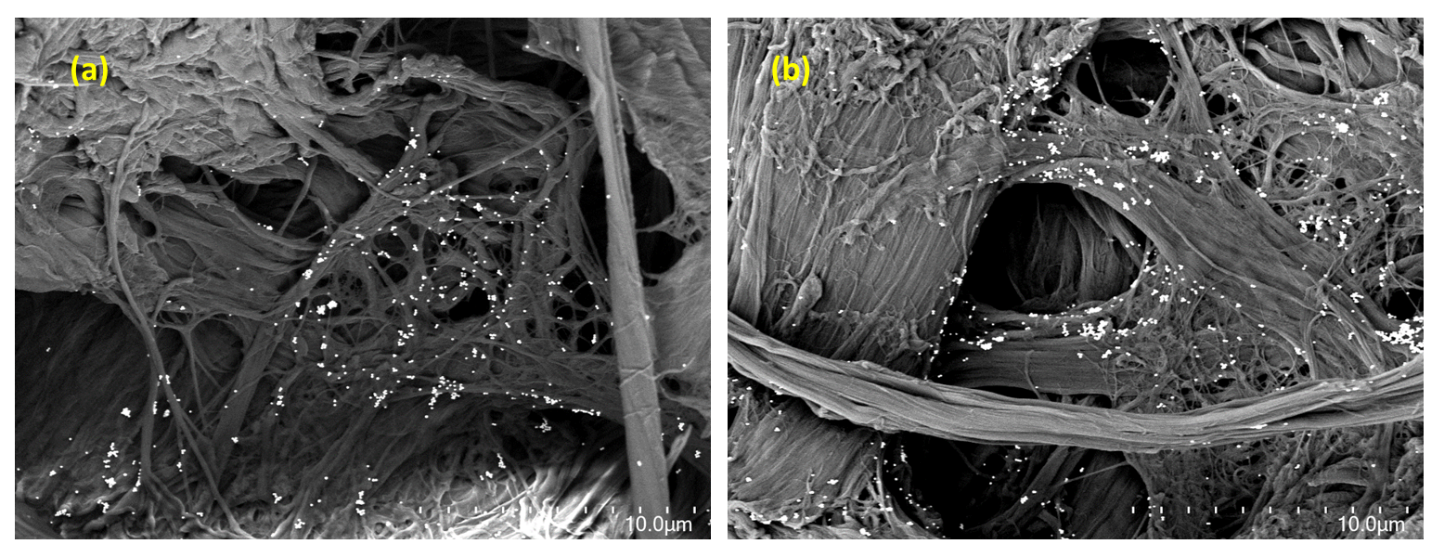


# Batch uniformity of the inkjet-printed sensors

Fig. S3. Three batches (A, B and C) of sensors were prepared following the same inkjet-printing process as described in the experimental section. Two sensors from each printing batch were randomly selected and exposed to 1 μM BPE. Ten spectra were measured from 10 random spots on each sensor. The integrated intensity of the 1204 cm^-1^ band is shown in the histogram. The green dashed line and the yellow shaded area indicate the mean and a relative standard deviation (RSD) of 12%. This indicates reasonable batch-to-batch reproducibility. All measurements were acquired with the handheld Raman analyzer.


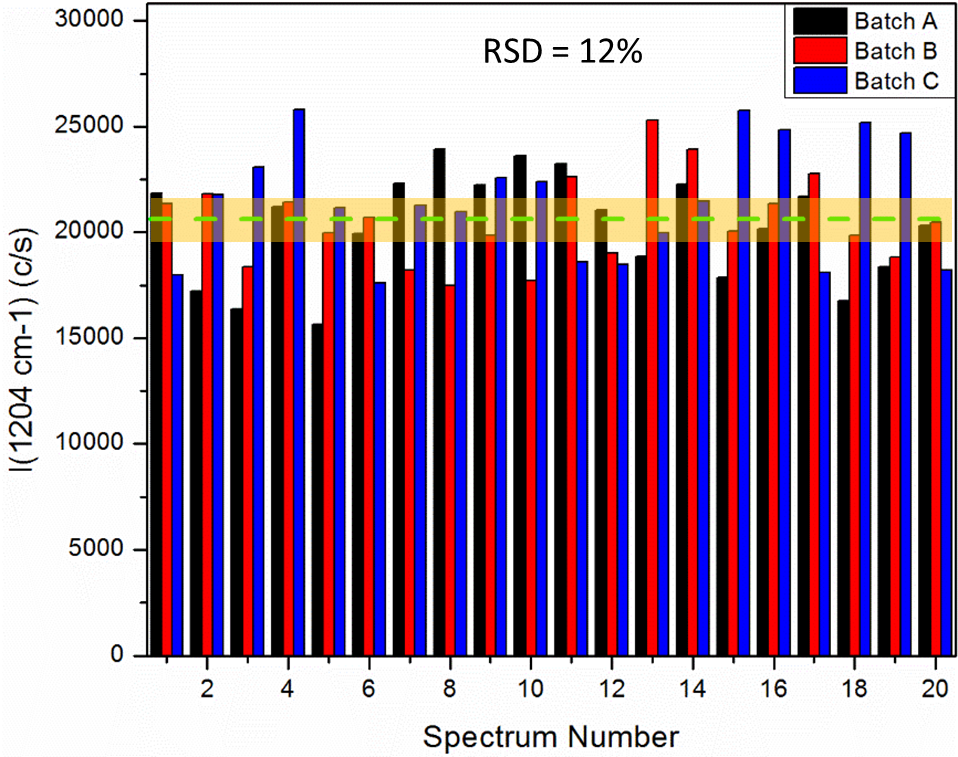


# Detection limit of Heroin with KI treated SERS sensors

Figure S4. (A) SERS sensors first functionalized with 10 mM KI followed by treatment of various concentrations of heroin. (B). Sensors functionalized with 20 mM KI followed by exposure to the same heroin concentrations. Heroin concentration and corresponding spectral trace colour are100 µg/mL (black), 10 µg/mL (red), 1µg/mL (blue), 100 ng/mL(magenta), 10 ng/mL (green), 1 ng/mL (navy). Red arrows indicate the heroin vibration at 623 cm^-1^. All spectra have been offset for clarity. The tick increment on the y-axis is 2000 counts. Note that in both cases, the detection limit is 10 ng/mL.


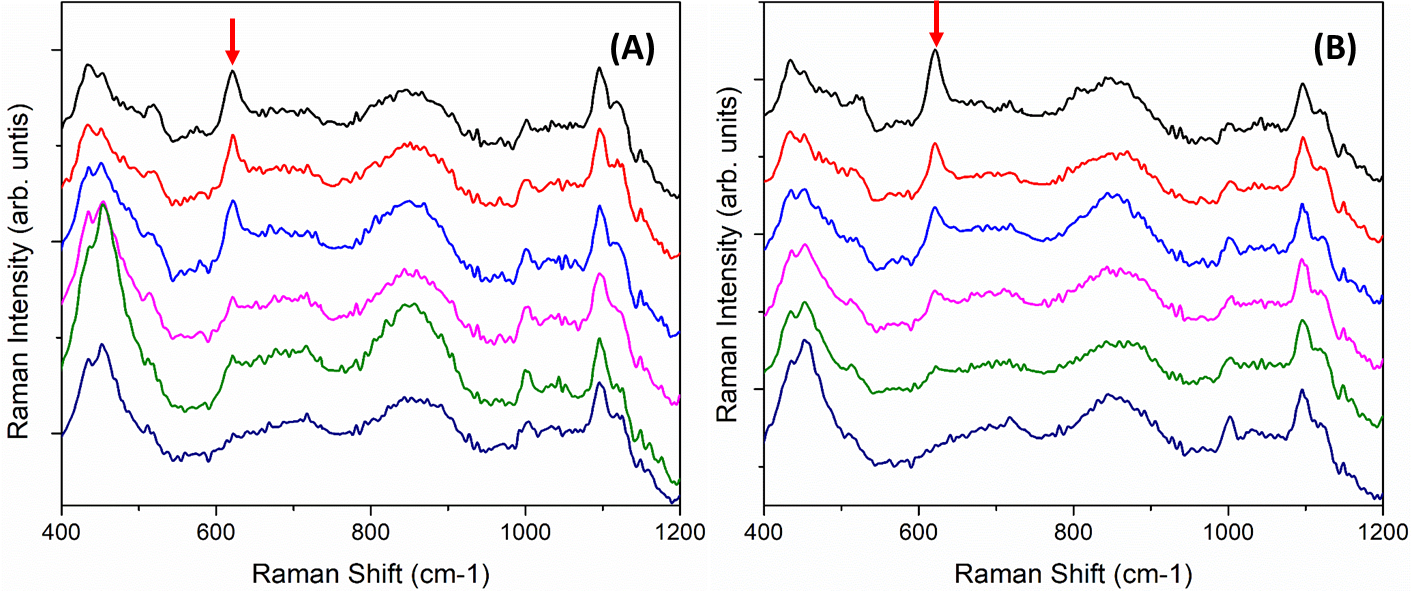

Supplement: Supplementary file 1 [file DataSheet1.docx]
